# Mechanistic evidence for dibutyl phthalate as an environmental trigger for inflammatory bowel disease

**DOI:** 10.1016/j.isci.2025.114619

**Published:** 2026-01-05

**Authors:** Hang Yuan, Gang Chen, Xuejun Sun, Junhui Yu

**Affiliations:** 1 Department of General Surgery, The First Affiliated Hospital of Xi’an Jiaotong University, Xi’an 710061, China; 2 The First Affiliated Hospital, and College of Clinical Medicine of Henan University of Science and Technology, Luoyang 471003, China

**Keywords:** toxicology, environmental toxicology

## Abstract

Dibutyl phthalate (DBP) is a ubiquitous pollutant, but its molecular link to inflammatory bowel disease (IBD) is undefined. We employed an integrative network toxicology framework, combining DBP target databases with IBD patient transcriptomics to address this gap. A computational pipeline using machine learning and molecular docking predicted a core six-gene signature (KYNU, PCK1, LCN2, CDC25B, EPHB4, SORD). We validated these predictions in human colonic epithelial cells (NCM460). DBP exposure induced a pro-inflammatory state and upregulated the core genes, with LCN2 showing the strongest response. Crucially, siRNA-mediated silencing of LCN2 significantly attenuated the DBP-induced inflammatory response, establishing it as a key functional mediator. Our findings provide direct mechanistic insight into how DBP can promote intestinal inflammation. The functional validation of LCN2 confirms the predictive power of our integrated approach and identifies a panel of target genes for future IBD investigation and environmental risk assessment.

## Highlights

• Network toxicology links dibutyl phthalate (DBP) to IBD molecular mechanisms
• DBP perturbs pathways of inflammation, lipid metabolism, and matrix remodeling
• Silencing the core target LCN2 attenuates DBP-induced inflammation in colon cells
• The findings provide a basis for the environmental risk assessment of DBP in IBD

## Graphical abstract

**Figure:**
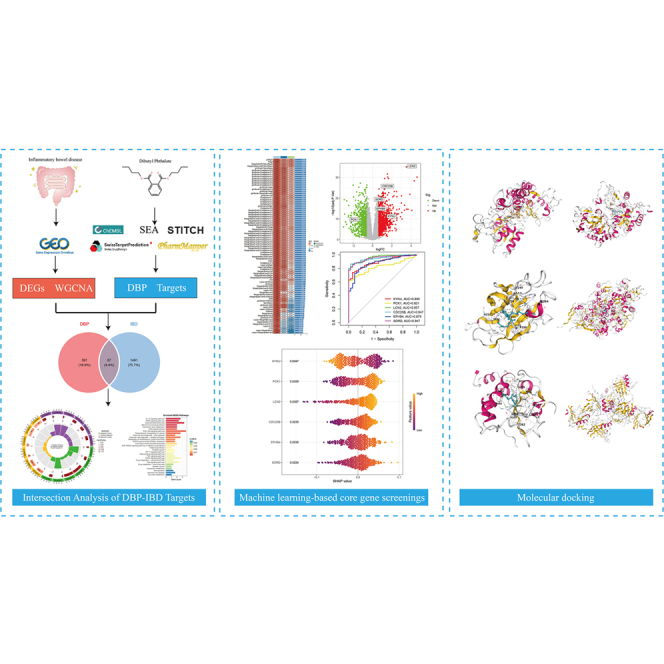

## Introduction

Emissions from industrial and agricultural activities have rendered phthalate esters (PAEs) ubiquitous environmental pollutants. They are now reported in water, air, and soil compartments,^1^^,^^2^^,^^3^^,^^4^ with accumulation documented in agricultural commodities.^5^^,^^6^ Among them, dibutyl phthalate (DBP)—a high-production-volume PAE—is consistently measured in human biological samples.^7^ Its extensive incorporation into consumer products has raised concerns about multi-organ toxicities affecting the reproductive, immune, and hepatic systems.^2^^,^^8^^,^^9^ Human exposure occurs via inhalation, ingestion, and dermal contact.^10^

Inflammatory bowel disease (IBD), which includes Crohn’s disease (CD) and ulcerative colitis (UC), is a chronic, immune-driven disorder that predominantly affects the gastrointestinal tract. The condition emerges from intricate gene-environment interactions, and chemicals such as DBP may modify disease risk or course through several exposure routes, including the consumption of contaminated water, dietary uptake through aquatic organisms, and microbiome-mediated effects.^11^ Reinforcing its potential role, recent work by Xiong et al. demonstrated that oral DBP administration in mice directly causes gut inflammation, disrupts the mucosal barrier, and alters microbial homeostasis.^12^ Although environmental pollutants have been broadly linked to IBD, the initiating molecular events by which DBP could shape IBD development remain insufficiently characterized.

In view of the complete lack of mechanistic studies on DBP-IBD interactions, we implemented an integrative network toxicology framework to probe potential biological pathways through which DBP might influence IBD progression. This approach fuses multi-omics datasets, network dynamics modeling, and in silico structure-activity relationship analyses to interrogate complex toxicant-host interactions and deepen mechanistic understanding. In parallel, network topological analyses, functional enrichment profiling, and machine-learning algorithms were used to identify high-priority hub molecules. Ensemble molecular docking was subsequently employed to validate target-binding affinities and interaction thermodynamic profiles. By charting a DBP-associated multiscale IBD-relevant molecular network, this study reveals candidate therapeutic targets in the context of DBP exposure. It further advances insight into how environmental pollutants contribute to IBD-relevant mechanisms, offering valuable perspectives to inform environmental health policy and guide future IBD research.

## Results

### Characterization of dibutyl phthalate-binding proteins

**Figure 1:**
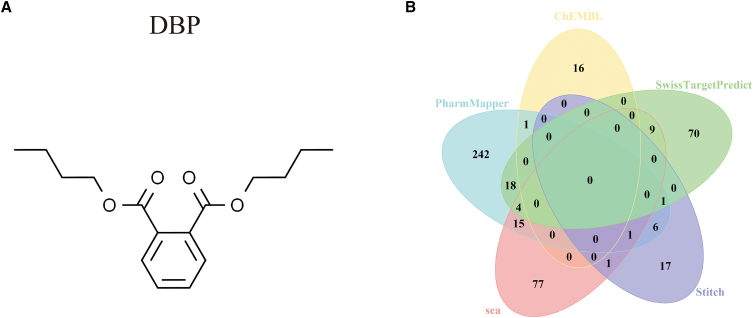
Characterization of DBP-interacting proteins (A) Depiction of DBP’s molecular configuration. (B) Target identification via integrated computational platforms: CHEMBI, SEA, SwissTargetPrediction, PharmMapper, and STITCH.

DBP was structurally annotated using PubChem (Figure 1A). Putative human protein targets were compiled by integrating outputs from five platforms: ChEMBL (bioactive compound curation), PharmMapper (reverse-pharmacophore mapping), SwissTargetPrediction (ligand-based inference), the SEA Search Server (Similarity Ensemble Approach), and STITCH. After merging results and removing duplicates, 478 unique candidate targets were retained (Figure 1B).

### Screening of inflammatory bowel disease-associated genes

**Figure 2:**
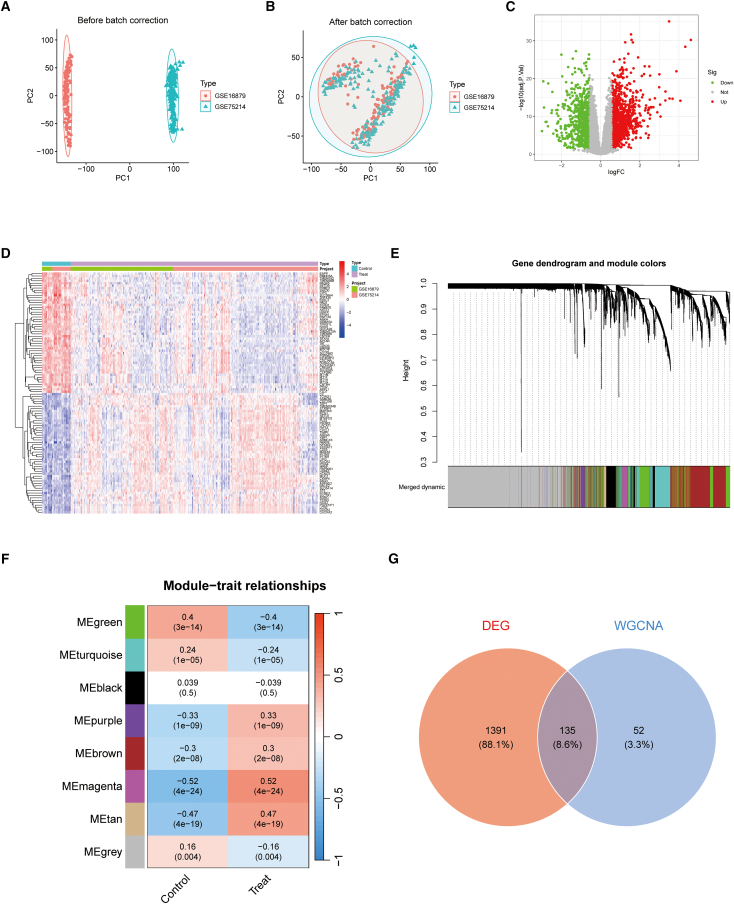
IBD-linked gene identification (A) Pre-correction PCA dispersion reveals batch effects between GSE16879 and GSE75214 cohorts. (B) Post-correction PCA demonstrates successful dataset integration with mitigated batch artifacts. (C) Volcano distribution of DEGs: Red = upregulated, green = downregulated, grey = nonsignificant (log_2_FC vs. significance). (D) Expression heatmap: red/blue denote up/down-regulated DEGs across specimens. (E) WGCNA dendrogram: Hierarchical gene clustering with color-coded co-expression modules. (F) Module-trait correlation heatmap: Associations between WGCNA modules and clinical status (control/treatment), annotated with correlation coefficients and P-values. (G) Intersectional genes: Venn diagram contrasting DEGs (red) and WGCNA modules (blue), with overlap indicating consensus targets.

Integrated transcriptomic datasets (GSE16879 and GSE75214) underwent batch-effect correction and normalization, after which PCA demonstrated clearer separation of sample clusters (Figures 2A and 2B). Differential expression analysis identified 1,526 significantly dysregulated genes in IBD (Figures 2C and 2D). For WGCNA, systematic evaluation of soft-thresholding powers (β = 1–20) indicated that β = 5 was the lowest value satisfying the scale-free topology criterion (R2≥0.80). A TOM-based hierarchical clustering then delineated eight co-expression modules (Figure 2E). Module-trait analysis identified modules significantly associated with IBD (*p* < 0.05; Figure 2F). Combining the DEG list with genes from IBD-associated modules (union, with redundancy removed) yielded 1,578 IBD-related genes (Figure 2G).

### Intersection analysis of dibutyl phthalate-inflammatory bowel disease targets

**Figure 3:**
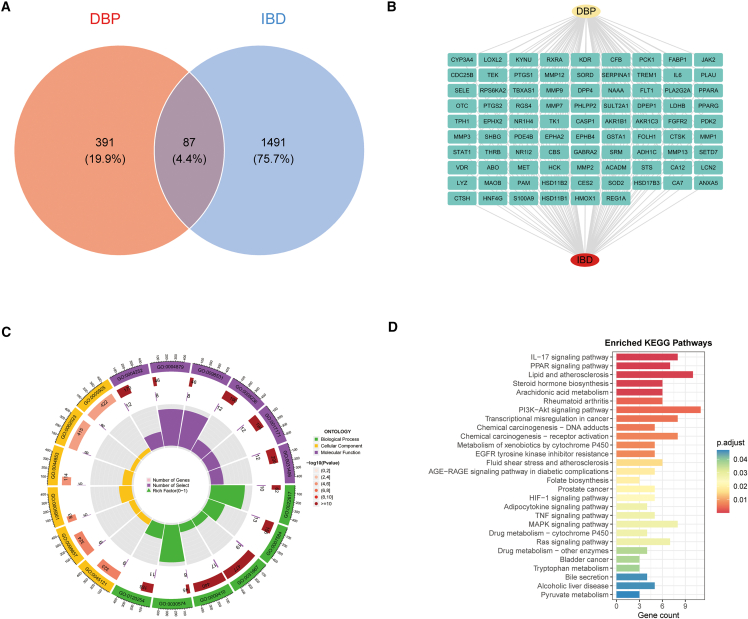
DBP-IBD target convergence (A) Venn analysis: DBP-associated genes (red) versus IBD-implicated genes (blue). (B) Protein-protein interaction network of consolidated DBP-IBD targets. (C) GO functional annotation: Bar plot of enriched terms across biological processes (BP), cellular components (CCs), and molecular functions (MFs). Bar length = gene count; color intensity = −log_10_ (adjusted P). (D) KEGG pathway enrichment: Dot plot shows pathway significance. Dot size = gene count; color gradient = −log_10_(adjusted P); x axis = gene ratio.

In the analysis of DBP-targeted IBD proteins/genes, we uncovered 87 pivotal targets driving DBP-affected IBD progression (Figure 3A). A DBP-IBD-target triad network was then established (Figure 3B). This network allowed for GO/KEGG enrichment, which delivered mechanistic insights into these targets (Figures 3C and 3D). Gene Ontology (GO) analysis of the DBP-IBD gene set revealed significant enrichment across biological process (BP), cellular component (CC), and molecular function (MF) categories (FDR-adjusted *p* < 0.05). BP terms were dominated by environmental stress and metabolic responses together with extracellular matrix (ECM) remodeling, including responses to nutrients, xenobiotics, chemical stress, and hypoxia, fatty-acid/olefinic-compound metabolism, and ECM disassembly/collagen catabolism. CC terms localized gene products to membrane microdomains and adhesion interfaces—membrane raft/microdomain/caveolae and focal adhesion/cell-substrate junction—as well as to the collagen-containing ECM; additional signals were observed for endosomal lumen and peptidase-inhibitor complexes. MF enrichment highlighted serine-type peptidase/hydrolase activity, organic-acid binding, nuclear-receptor/ligand-activated transcription-factor activity, and protein tyrosine kinase activities.

KEGG pathway enrichment analysis identified significant over-representation of pathways (FDR<0.05) that converged on four themes: (1) inflammatory and stress signaling (IL-17, TNF, MAPK, PI3K-Akt, HIF-1, Ras, AGE-RAGE); (2) lipid and energy metabolism (PPAR signaling; lipid and atherosclerosis; arachidonic-acid and steroid-hormone metabolism; adipocytokine signaling; bile secretion; pyruvate and tryptophan metabolism); (3) xenobiotic/drug metabolism (cytochrome P450-mediated metabolism and chemical carcinogenesis modules); and (4) cancer-related signaling and resistance (EGFR-TKI resistance and transcriptional misregulation). By gene count, lipid and atherosclerosis, PI3K-Akt, and IL-17 signaling ranked among the most prominent.

Collectively, the enrichment patterns indicate that DBP-IBD-associated genes are primarily involved in inflammatory/stress signaling, lipid and xenobiotic metabolism, ECM remodeling, and membrane-raft/focal-adhesion-related cellular localization.

### Prioritization of core driver genes

**Figure 4:**
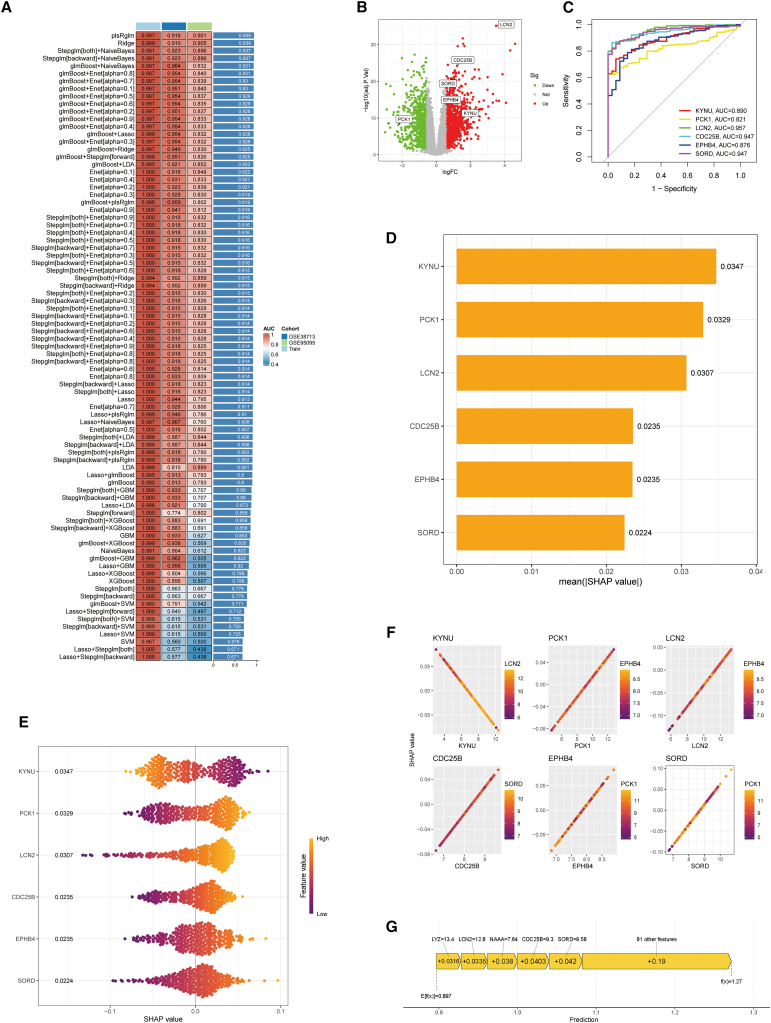
Core gene discovery in DBP-triggered IBD (A) Model efficacy comparison: Heatmap of AUC metrics across validation cohorts. Models (rows) versus performance (columns), color-coded by cohort source. (B) Differential expression landscape: Volcano plot highlights DEGs (labeled key genes; red/green = up/down-regulation). (C) Diagnostic ROC curves: Performance evaluation of pivotal genes (KYNU, PCK1, LCN2, CDC25B, EPHB4, and SORD) via sensitivity vs. 1-specificity. AUC values quantify predictive accuracy. (D) Feature prioritization: Bar plot ranking genes by model contribution magnitude. (E) Expression distribution: Violin plots contrast gene expression across experimental conditions. (F) SHAP dependence plot. The x axis shows gene expression, and the y axis shows the SHAP value for the model output (higher values push the prediction toward IBD). Each point represents one sample, and the color indicates the expression level of a potential interacting feature. (G) SHAP interpretability: Summary plot delineating directional impact of genes on predictions (negative/positive SHAP = protective/risk effects).

We screened 87 candidates with multiple classifiers and identified a PLS-GLM-based ensemble as the top performer (Figure 4A). The ensemble achieved an internal cross-validated AUC of 0.997 and validated well in two independent cohorts (GSE38713 AUC = 0.918; GSE95095 AUC = 0.901; mean external AUC = 0.939). Stability of feature importance across resamples prioritized six genes: KYNU, PCK1, LCN2, CDC25B, EPHB4, and SORD. As single markers, each gene showed good discrimination (AUCs: LCN2 0.957, CDC25B 0.947, SORD 0.947, KYNU 0.890, EPHB4 0.876, PCK1 0.821; Figure 4C). Differential expression indicated that LCN2, CDC25B, SORD, EPHB4, and KYNU were upregulated in IBD mucosa, whereas PCK1 was downregulated (Figure 4B). SHAP analyses ranked KYNU and PCK1 among the most influential features (mean|SHAP| = 0.0347 and 0.0329, respectively; Figure 4D). Dependence plots were approximately linear, showing positive effects for LCN2, CDC25B, EPHB4, SORD, and PCK1, and a negative effect for KYNU (Figure 4E). Notably, PCK1 is downregulated in IBD despite its positive model effect, suggesting a conditional, multivariable influence. These directions indicate conditional, multivariable effects and likely reflect feature correlations. Representative waterfall plots illustrate per-sample contributions of the six genes together with other features (Figures 4F and 4G). Overall, LCN2, CDC25B, EPHB4, and SORD contribute positively to IBD classification, whereas KYNU and PCK1 exhibit context-dependent effects.

### Molecular docking of dibutyl phthalate-core gene interactions

**Figure 5:**
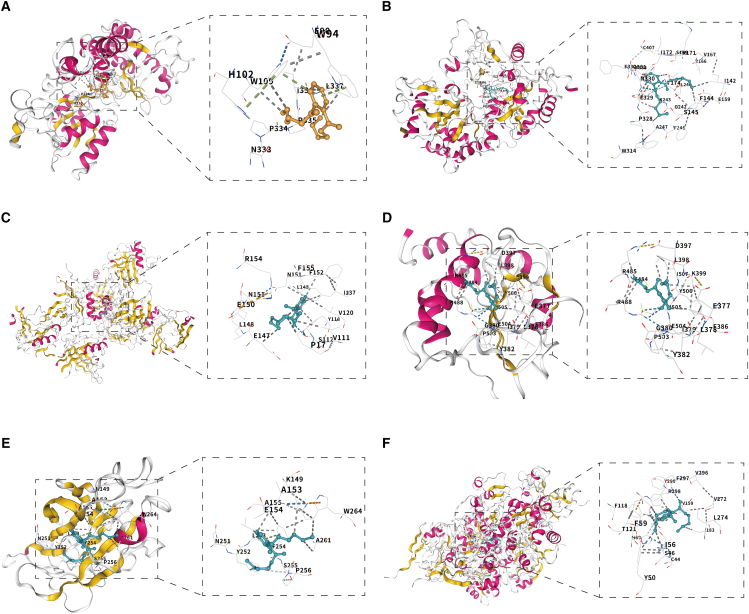
Computational docking of DBP with core gene products: (A–F) Structural binding analyses between DBP and KYNU (A), PCK1 (B), LCN2 (C), CDC25B (D), EPHB4 (E), and SORD (F)

To examine DBP interactions with six principal targets (KYNU, PCK1, LCN2, CDC25B, EPHB4, and SORD), molecular docking analyses were carried out on the CB-Dock2 platform. The predicted binding energies ranged from −5.3 to −6.9 kcal/mol (Table 1). All values were more favorable than the commonly used −5.0 kcal/mol benchmark that typically denotes strong ligand-protein affinity.^13^ The negative binding energies (<0 kcal/mol) further indicate that complex formation with each target is thermodynamically spontaneous. Taken together, these characteristics suggest that the identified interactions could contribute to DBP-mediated IBD. Representative three-dimensional docking poses for these complexes are presented in Figures 5A–5E.

**Table 1:** Binding energies of ligands and receptors

Ligand	Receptor	Bind. Energy[kcal/mol]
DBP	KYNU	−5.3
DBP	PCK1	−6.9
DBP	LCN2	−6.5
DBP	CDC25B	−5.6
DBP	EPHB4	−5.6
DBP	SORD	−6.6

### Dibutyl phthalate exposure triggers a pro-inflammatory response in colonic epithelial cells via the upregulation of LCN2

**Figure 6:**
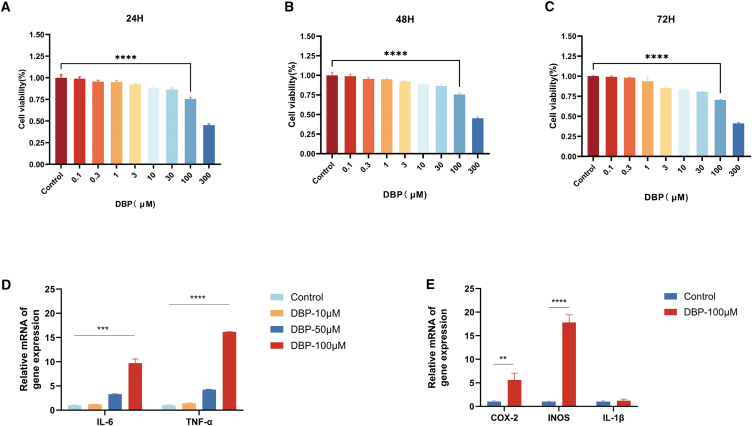
DBP induces a pro-inflammatory response in NCM460 colon epithelial cells at sub-cytotoxic concentrations (A–C) Effects of different concentrations of DBP on the viability of human normal colon epithelial NCM460 cells at 24, 48, and 72 h, as determined by the CCK-8 assay (*n* = 3). (D) qRT-PCR analysis of IL-6 and TNF-α in NCM460 cells after 48 h of treatment with increasing concentrations of DBP (*n* = 3). (E) qRT-PCR analysis of COX-2, iNOS, and IL-1β in NCM460 cells after 48 h of treatment with 100 μM DBP (*n* = 3). ∗*p* < 0.05; ∗∗*p* < 0.01; ∗∗∗*p* < 0.001; ∗∗∗∗*p* < 0.0001.

To determine a suitable concentration of DBP for our study, we first assessed its cytotoxicity in normal human colonic epithelial cells. A CCK-8 assay with a wide range of concentrations (0.1–300 μM) showed that doses up to 100 μM caused only moderate (∼20%) inhibition of cell proliferation after 48 h Figures 6A–6C). We then performed a dose-response analysis (0, 10, 50, 100 μM) and determined that 100 μM was the lowest concentration to significantly increase the expression of the key inflammatory markers TNF-α and IL-6 (Figure 6D). Based on these results, 100 μM was chosen for all further experiments. At this concentration, DBP induced a broad inflammatory state, significantly upregulating not only TNF-α and IL-6 but also other pro-inflammatory mediators such as iNOS and COX-2 (Figure 6E).

**Figure 7:**
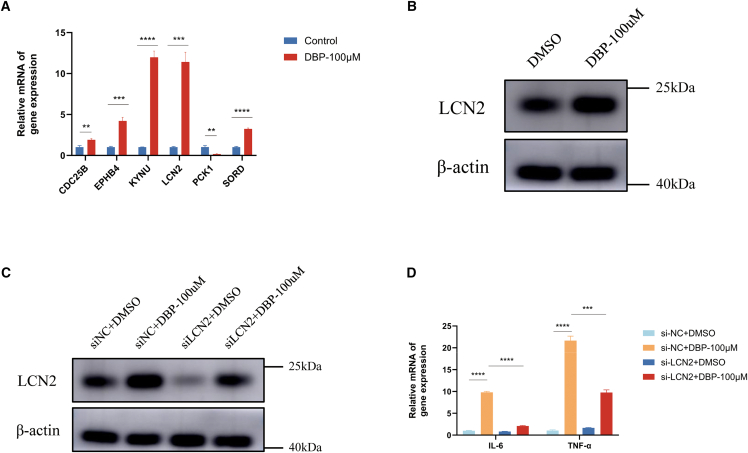
LCN2 mediates DBP-induced inflammation in NCM460 Colon Epithelial Cells (A) qRT-PCR analysis of target gene (KYNU, PCK1, LCN2, CDC25B, EPHB4, and SORD) expression in NCM460 cells after 48 h of treatment with 100 μM DBP (*n* = 3). (B) Western blot analysis of LCN2 protein expression in NCM460 cells after 48 h of treatment with 100 μM DBP. (C and D) For knockdown experiments, cells were transfected with control (si-NC) or LCN2-targeting siRNA (si-LCN2#1) for 24 h, followed by treatment with DBP (100 μM) or vehicle (DMSO) for 48 h. (C) Western blot confirms efficient LCN2 protein knockdown. (D) qRT-PCR analysis shows that LCN2 silencing attenuated the DBP-induced expression of IL-6 and TNF-α (*n* = 3). ∗*p* < 0.05; ∗∗*p* < 0.01; ∗∗∗*p* < 0.001; ∗∗∗∗*p* < 0.0001.

To further elucidate the core molecular drivers underlying this DBP-induced inflammation, we next validated the expression of six hub genes previously identified through machine learning-based bioinformatics prediction. Notably, the expression trends of all six genes were experimentally confirmed to be consistent with the bioinformatics predictions, underscoring the reliability of our screening model (Figure 7A). Among these candidates, LCN2 emerged as one of the most significantly upregulated genes following DBP stimulation. Given its well-established critical role in the pathogenesis of IBD, LCN2 was prioritized for subsequent investigation. We confirmed that this transcriptional increase was mirrored at the protein level, as Western blot analysis showed that DBP treatment markedly elevated LCN2 protein expression in NCM460 cells (Figure 7B). These results identify LCN2 as a key protein robustly induced by DBP at both the mRNA and protein levels.

To determine if the upregulation of LCN2 was functionally involved in the pro-inflammatory response, we used siRNA to knock down its expression before DBP exposure. We first confirmed the knockdown efficiency of two distinct siRNAs, selecting the more effective one (si-LCN2#1) for subsequent experiments (Figure S1). Critically, in LCN2-knockdown cells, the DBP-induced upregulation of the key inflammatory cytokines IL-6 and TNF-α was significantly attenuated compared to control cells (si-NC) treated with DBP (Figures 7C and 7D). Collectively, these findings demonstrate that LCN2 is not merely a marker of DBP-induced inflammation but acts as a critical functional mediator of this pro-inflammatory process in colonic epithelial cells.

## Discussion

Our integrative network toxicology analysis identified a robust six-gene signature (KYNU, PCK1, LCN2, CDC25B, EPHB4, SORD) that mechanistically links DBP exposure to IBD biology. This signature was derived from an initial pool of 87 candidates using an unbiased, multi-algorithm ensemble approach, and its reliability is underscored by several key lines of evidence. First, its high diagnostic performance (AUC >0.90) was rigorously validated in two independent external cohorts. Second, SHAP analysis provided model interpretability, revealing KYNU, PCK1, and LCN2 as the most informative predictors. Third, molecular docking predicted plausible direct binding of DBP to all six proteins with favorable energies (−5.3 to −6.9 kcal/mol). Finally, and most critically, we grounded these computational findings with *in vitro* experiments, demonstrating that the expression of all six genes in human colonic cells responded to DBP exposure exactly as predicted. This multi-pronged validation establishes our signature as a reliable molecular link between an environmental toxicant and IBD pathogenesis.

The biological importance of this six-gene signature is reinforced by a broader pathway enrichment analysis of all DBP-related targets, which highlighted three interconnected themes central to IBD. First, DBP engages a network of inflammatory and hypoxic stress responses, including critical pathways such as IL-17, TNF, and HIF-1 that mediate immune activation and adaptation to the inflamed gut microenvironment.^14^^,^^15^^,^^16^^,^^17^^,^^18^^,^^19^^,^^20^^,^^21^ Second, it impacts metabolic regulation, particularly through nuclear receptors (PPARs) and cytochrome P450 enzymes, linking lipid and xenobiotic metabolism directly to inflammatory control.^22^^,^^23^^,^^24^^,^^25^ Finally, these processes are coupled with the dysregulation of extracellular matrix remodeling and cell adhesion, critical for maintaining epithelial barrier integrity and preventing maladaptive repair.^26^^,^^27^^,^^28^^,^^29^ This broader analysis provides a mechanistic landscape that validates the biological domains represented by our core signature.

Functionally, the six genes cluster into three interconnected groups:(1) Metabolism and redox balance: KYNU, a key enzyme in the tryptophan-kynurenine pathway, modulates immune responses and oxidative stress by regulating NAD+ metabolism, thereby influencing epithelial resilience and inflammation in IBD.^30^^,^^31^^,^^32^ PCK1, involved in gluconeogenesis and glyceroneogenesis, plays a crucial role in maintaining cellular energy homeostasis and epithelial barrier integrity during intestinal inflammation.^33^^,^^34^^,^^35^ SORD, which participates in the polyol pathway, affects cellular NADH/NAD+ balance and oxidative stress, further contributing to epithelial protection and immune modulation.^36^^,^^37^ (2) Innate immunity and barrier defense: LCN2 supports iron sequestration, thus limiting microbial growth, and provides mucosal protection, aligning closely with neutrophil activity characteristic of IBD inflammation.^38^^,^^39^^,^^40^ LCN2’s elevated expression is also associated with intestinal inflammation and has been recognized as a biomarker for mucosal damage and colorectal cancer risk in IBD. (3) Cell growth and tissue architecture: CDC25B facilitates the G2/M cell-cycle transition, promoting cell proliferation necessary for epithelial repair processes, while EPHB4 regulates epithelial compartmentalization and angiogenesis, essential for tissue homeostasis and remodeling.^41^^,^^42^^,^^43^ Both genes play roles in tissue repair and have implications in malignant transformation during chronic inflammation.

To bridge our computational predictions with biological reality, we performed *in vitro* validation using NCM460 cells. This exposure induced a significant pro-inflammatory response, evidenced by the upregulation of mediators such as IL-6 and TNF-α. Our experiments validated the expression of all six core genes, with LCN2 showing the most pronounced upregulation at both the transcriptional and translational levels. The significance of this finding was further underscored by functional experiments. Using siRNA, we demonstrated that knockdown of LCN2 significantly blunted the DBP-induced inflammatory response, confirming that LCN2 acts as a key downstream mediator. This provides direct experimental evidence for a DBP-LCN2-inflammation axis. Given that LCN2 is a key player in innate immunity and a recognized biomarker for IBD activity, our findings reinforce the central hypothesis that DBP can actively contribute to intestinal inflammation.

For this study, we used a DBP concentration of 100 μM, which was determined by dose-response experiments to be the lowest effective concentration to elicit a significant pro-inflammatory response without causing overt cytotoxicity. While this concentration exceeds the average daily exposure levels for the general population (7–10 μg/kg/day),^44^^,^^45^^,^^46^ it is highly relevant for modeling the “first-hit” exposure of gut epithelial cells, which can experience much higher transient local concentrations following ingestion.^47^^,^^48^ Furthermore, this concentration is consistent with the 10–100 μM range used in many published phthalate studies, ensuring our findings are comparable to existing literature.^44^^,^^49^^,^^50^^,^^51^

A critical distinction in phthalate toxicology is the separate roles of the parent compound and its metabolites. While monobutyl phthalate (MBP) is the key mediator of systemic toxicity, our study was designed to establish the direct toxicity of the parent DBP compound at the intestinal barrier—the “primary insult” of oral exposure. Our results support this hypothesis, demonstrating that DBP is itself a primary pathogenic trigger of gut inflammation. This implicates the parent compound as a key initiator of local toxicity, providing a mechanistic basis to explore the cascade of systemic effects later mediated by MBP. This approach allows for a clearer differentiation between the initiating local toxicity of DBP and the subsequent systemic effects of its metabolite.

Our findings also open several avenues for future translational research. First, our results support the implementation of biomonitoring for both DBP exposure and our six-gene signature to identify at-risk populations. Second, the highlighted pathways—metabolic reprogramming, cell cycle, and innate immunity—present clear targets for drug repurposing strategies. Third, our gene panel could be developed into a companion diagnostic for risk stratification or therapeutic response tracking. On a larger scale, integrating our molecular data with systems pharmacology models could help design and test multi-target interventions to mitigate the impact of environmental exposures on IBD.

This study demonstrates that DBP contributes to intestinal inflammation by modulating a core set of molecular targets, with LCN2 acting as a key mediator. Our integrated workflow, combining in silico prediction and experimental validation, successfully identified these targets and confirmed their functional role in the DBP-induced pro-inflammatory response. These results provide a validated mechanistic framework for understanding DBP’s impact on IBD and identify LCN2 as a promising candidate for further research and potential therapeutic intervention.

### Limitations of the study

A primary limitation of this study is its reliance on an *in vitro* monoculture model. While this approach was essential for isolating the direct epithelial response to DBP, it inherently lacks the complexity of the *in vivo* IBD microenvironment, which involves dynamic interactions between epithelial cells, immune cells, and the gut microbiome. Therefore, our findings represent a foundational step that requires validation in more physiologically relevant systems. Future work should progress toward co-culture or intestinal organoid models, and ultimately to *in vivo* validation using animal models, such as DSS-induced colitis, with environmentally relevant DBP exposure. Such studies are crucial to confirm the expression of the six-gene signature (KYNU, PCK1, LCN2, CDC25B, EPHB4, SORD) in colonic tissue and directly link our molecular hypothesis to IBD pathology.

Second, our molecular docking results are predictive and only suggest structural plausibility for DBP’s interaction with target proteins. They do not confirm binding or functional consequences. To build upon this structural hypothesis, further validation is essential. Computationally, molecular dynamics (MD) simulations should be employed to assess the stability of the predicted binding poses. Experimentally, biophysical assays, such as Surface Plasmon Resonance (SPR), are needed to confirm direct binding. Furthermore, functional assays—for instance, enzyme activity assays for KYNU and PCK1—are required to determine the functional impact of this interaction. Ultimately, gain- and loss-of-function studies will be necessary to establish a causal link between DBP binding and cellular outcomes.

Finally, the foundational association link between DBP and the IBD transcriptome was inferred from an integrative analysis rather than measured directly in patient cohorts. Consequently, our findings establish a robust molecular hypothesis for DBP’s potential role in IBD, but do not demonstrate causality at the population level. Direct epidemiological studies linking specific DBP exposure to IBD development are needed to bridge this critical gap.

## Resource availability

### Lead contact

Requests for further information and resources should be directed to and will be fulfilled by the lead contact, Junhui Yu (yujunhui@mail.xjtu.edu.cn).

### Materials availability

All materials generated in this study will be available upon request.

### Data and code availability

The data related to GO and KEGG analysis reported in this article can be accessed from the Gene Expression Omnibus (GEO: https://www.ncbi.nlm.nih.gov/geo/). The structural data for the molecular docking of DBP-bound targets are available in the Protein DataBank (PDB) under the following accession codes: KYNU (7S3V), PCK1 (1KHF), LCN2 (3BX8), CDC25B (1CWR), EPHB4(2BBA), and SORD (9H92). The authors confirm that all data underlying the findings are fully available without restriction. All relevant data are within the article.
This article does not report original code.
Any additional information required to reanalyze the data reported in this article is available from the lead contact upon request.

## Acknowledgments

None.

## Author contributions

Xuejun Sun and Junhui Yu contributed to conceptualization, formal analysis, funding acquisition, and project administration; Hang Yuan performed data curation, investigation, resources validation, and supervision; Gang Chen contributed to software and visualization; Hang Yuan and Gang Chen were involved in methodology; Hang Yuan and Junhui Yu contributed to writing - original draft and writing - review and editing. All authors approved the final version of the article, including the authorship list.

## Declaration of interests

The authors declare no competing interests.

## STAR★Methods

### Key resources table

**Table:** 

REAGENT or RESOURCE	SOURCE	IDENTIFIER
**Antibodies**		

LCN2	Selleck	Cat#F0999
β-actin	Proteintech	Cat#81115-1-RR

**Chemicals, peptides, and recombinant proteins**		

Dibutyl phthalate	MCE	Cat#HY-Y0304

**Critical commercial assays**		

Lipo8000™ Transfection Reagent	Beyotime	Cat#C0533
Cell Counting Kit-8	TargetMol	Cat#C0005
Total RNA Extraction Kit	TIANGEN	Cat#DP419
StarScript III RT MasterMix	GenStar	Cat# A233-10

**Deposited data**		

Raw and analyzed data	GEO database	GSE16879, GSE75214, GSE38713, GSE95095

**Oligonucleotides**		

siRNA targeting sequence: LCN2 #1: GCUGGGCAACAUUAAGAGUUAdTdT	This paper	N/A
siRNA targeting sequence: LCN2 #2: CCAGCAUGCUAUGGUGUUCUUdTdT	This paper	N/A
Primers for all qPCR, see Table S1	This paper	N/A

**Software and algorithms**		

GraphPad Prism 10 software	GraphPad Software	http://www.graphpad.com/
R software	R software (v4.3.1)	https://www.r-project.org

### Experimental model and study participant details

The human normal colon epithelial cell line, NCM460, was procured from the Chinese Academy of Sciences Cell Bank located in Shanghai, China. The cell line was authenticated by short tandem repeat (STR) profiling and tested for mycoplasma contamination. These cells were maintained under standard culture conditions in a humidified incubator at 37°C with an atmosphere of 5% CO_2_. For the experimental treatments, the NCM460 cells were exposed to a medium containing 100μM DBP, while a 0.1% DMSO solution served as the vehicle control. No human subjects/samples were used in this study.

### Method details

#### Identification of pathogenesis-linked molecular targets

Transcriptomic datasets for IBD were acquired from the NCBI GEO database, specifically accessions GSE16879, GSE75214, GSE38713, and GSE95095. The investigation designated GSE16879 and GSE75214 as the discovery cohort, reserving GSE38713 and GSE95095 for independent validation. To mitigate batch effects, a three-phase normalization protocol was implemented: (1) Latent Variable Adjustment: Surrogate Variable Analysis (SVA) modeled and corrected undisclosed technical variations within the discovery cohort; (2) Cross-Dataset Harmonization: The ComBat algorithm employed parametric empirical Bayesian methods to align inter-study heterogeneity; (3) Validation Phase: Principal component analysis (PCA) confirmed enhanced sample stratification in reduced-dimensional space post-correction, verifying normalization efficacy.

#### Compound characterization and target profiling for DBP

A multi-source integration strategy was applied for DBP analysis: (1) Physicochemical Profiling: Systematic extraction of compound attributes through PubMed; (2) Structural Annotation: Acquisition of standardized 2D structural representations (SMILES: CCCCOC(=O)C1=CC=CC=C1C(=O)OCCCC) from PubChem; (3) Target Identification: Ligand-receptor interaction mapping using ChEMBL,^52^ chemical genomics-based predictions via SwissTargetPrediction,^53^ 3D pharmacophore alignment with PharmMapper,^54^ supplemented by SEA Search Server and STITCH database analyses.^55^^,^^56^ All predicted targets were exclusively restricted to Homo sapiens.

#### Differential expression profiling

Gene expression differentials were analyzed via the limma package, defining statistically significant differentially expressed genes (DEGs) by thresholds of adjusted p < 0.05 and |log2FC| > 0.585 (corresponding to 1.5-fold change). This methodology enabled detection of IBD progression- and treatment response-associated genes.

#### Weighted Co-Expression network Construction (WGCNA)

Co-expression networks were generated using WGCNA, initiating with sample quality assessment through hierarchical clustering to exclude outliers. Dynamic tree-cutting determined the soft-thresholding power (scale-free topology fit R^2^ ≥ 0.85). Modules were identified via hierarchical clustering of the topological overlap matrix (TOM) under parameters: minimum module size = 60, merge cut height = 0.25. Module-trait correlations were computed by eigengene-phenotype Pearson analysis (|r| > 0.5, p < 0.05), with hub genes selected by intramodular connectivity (kME > 0.8).

#### Integration of DBP-Associated disease targets

Intersection analysis of DEGs, WGCNA hub genes, and DBP-predicted targets identified pivotal DBP-IBD interaction targets, visualized through Venn diagrams to elucidate their pathological relevance.

#### Functional annotation

Gene Ontology (Biological Process; Cellular Component; Molecular Function) and KEGG pathway enrichment (p < 0.05) were conducted with clusterProfiler, revealing DBP's functional mechanisms in IBD pathogenesis.

#### Machine learning pipeline for diagnostic marker identification

Model training and feature selection were conducted on a discovery cohort (integrated GSE16879 and GSE75214; N = 255), while two independent cohorts, GSE38713 (N = 43) and GSE95095 (N = 60), were held out for final validation.

Within the discovery cohort, we compared eleven algorithms (Lasso, SVM, XGBoost, LDA, plsRglm, NaiveBayes, glmBoost, Stepglm, Ridge, Enet, GBM) using a stratified 5-fold cross-validation (80% training, 20% validation splits). Each model's performance was evaluated by its average Area Under the Curve (AUC), accuracy, and F1-score. Core markers were then identified by selecting genes that showed consistent importance across the top-performing models. To interpret the final model built from these markers, we used SHAP (Shapley Additive Explanations) to quantify how each gene impacted diagnostic predictions. The predictive power of this final gene signature was then assessed on the two independent validation cohorts.

#### Ligand-target binding validation

Ligand structures (SDF format) and target proteins (PDB) underwent structural minimization (hydrogen addition, water removal) in CB-Dock2. Binding poses were ranked by Vina score, with the lowest score indicating optimal affinity.

#### CCK-8

Cell viability was assessed using the Cell Counting Kit-8 (CCK-8; CAT#C0005, TargetMol, USA). Initially, NCM460 cells were plated into 96-well plates at a density of 4,000 cells per well. After a 24-hour incubation period, the existing medium was exchanged for fresh medium supplemented with various concentrations of DBP (HY-Y0304, MCE, USA). At 24, 48, and 72 hours post-treatment, CCK-8 reagent was introduced to each well to quantify cell proliferation. The plates were then incubated for an additional 2 hours at 37°C before the absorbance at 450 nm (OD450) was measured using a microplate reader.

#### RNA Interference

Small interfering RNAs (siRNAs) targeting human LCN2 (si-LCN2) and a non-targeting negative control (si-NC) were synthesized by Tsingke Biotechnology Co., Ltd. (Beijing, China). Lipo8000™ Transfection Reagent (C0533, Beyotime Biotechnology, Shanghai, China) was used to transfect the siRNAs into NCM460 cells according to the manufacturer's protocol.

#### Quantitative real-time PCR (qRT-PCR)

Following the manufacturer's protocol, total RNA was isolated from cells using the Total RNA Extraction Kit (DP419, TIANGEN, China). Subsequently, cDNA was synthesized from the extracted RNA through reverse transcription with the StarScript III RT MasterMix (A233-10, GenStar, China) in a 20 μl reaction volume. The quantitative real-time PCR amplification was carried out on a CFX96 Real-Time PCR Detection System (Bio-Rad, USA) using the RealStar Fast SYBR qPCR Mix (A301-5, GenStar, China). Gene expression levels were normalized to β-actin, which served as the internal reference gene. A complete list of primers used for this analysis is available in Table S1.

#### Western blot

Cellular proteins were extracted using RIPA lysis buffer (P0013B, Beyotime, Shanghai, China), and their concentrations were quantified with the BCA Protein Assay Kit (P0012, Beyotime, Shanghai, China). For each sample, an equal amount of protein was separated via SDS-PAGE and then transferred onto 0.22 μm PVDF membranes (Millipore). To prevent nonspecific binding, the membranes were blocked for one hour at room temperature with a solution of 5% non-fat milk in TBST. The membranes were subsequently probed overnight at 4°C with primary antibodies against LCN2 (F0999, Selleck, USA) and β-actin (81115-1-RR, Proteintech, USA). Following washes, the membranes were incubated for one hour at room temperature with the corresponding goat anti-rabbit IgG-HRP secondary antibody (abs20040, Absin, China). Protein bands were visualized using an ECL Chemiluminescence kit (MI00607B, Mishu Biotechnology, Xi'an, China).

### Quantification and statistical analysis

All experiments were performed with at least three independent biological replicates (n=3). All experimental results are presented as the mean ± standard error (SE). For statistical comparison, unpaired Student’s t-tests were utilized for two-group analyses, while one-way analysis of variance (ANOVA) was applied for multiple-group comparisons. All statistical computations were carried out using R software and GraphPad. The designated levels of statistical significance were ∗P < 0.05, ∗∗P < 0.01, ∗∗∗P < 0.001, and ∗∗∗∗P < 0.0001.
