# Supplementary material for: Mechanistic evidence for dibutyl phthalate as an environmental trigger for inflammatory bowel disease
Source: iScience. 2026 Jan 5;29(2):114619. doi: 10.1016/j.isci.2025.114619 (PMC12861011; doi:10.1016/j.isci.2025.114619)

iScience, Volume *29*

Supplemental information

Mechanistic evidence for dibutyl phthalate as an environmental trigger for inflammatory bowel disease

Hang Yuan, Gang Chen, Xuejun Sun, and Junhui Yu

Table S1: List of oligonucleotide sequences used in this study. Related to STAR Methods.

Name	Type	Species	Sequence (5'-3')
β-actin-F	Primer	Human	TGCGTGACATTAAGGAGAA
β-actin-R	Primer	Human	AAGGAAGGCTGGAAGAGT
IL-1β-F	Primer	Human	GGCTTATTACAGTGGCAATG
IL-1β-R	Primer	Human	GTAGTGGTGGTCGGAGAT
IL-6-F	Primer	Human	GTGAGGAACAAGCCAGAG
IL-6-R	Primer	Human	TGACCAGAAGAAGGAATGC
COX-2-F	Primer	Human	CGAGGTGTATGTATGAGTGT
COX-2-R	Primer	Human	AGCCATAGTCAGCATTGTAA
iNOS-F	Primer	Human	CAAGGCACAGGTCTCTTC
iNOS-R	Primer	Human	GCAGGTCACTTATGTCACT
TNF-α-F	Primer	Human	GCAACAAGACCACCACTT
TNF-α-R	Primer	Human	CTCCAGATTCCAGATGTCAG
LCN2-F	Primer	Human	CCTGGATTAACGAGTTACCT
LCN2-R	Primer	Human	TTGGAGAAGCGGATGAAG
CDC25B-F	Primer	Human	CTCCAGCCTGAACAGAAG
CDC25B-R	Primer	Human	ATCCATCCGCAACAAGAC
EPHB4-F	Primer	Human	GACAGTATCTCATCGGACAT
EPHB4-R	Primer	Human	CACCAATCACCTCTTCAATC
KYNU-F	Primer	Human	GGTTCCAAGTCTTCGCTAT
KYNU-R	Primer	Human	CCATCTCACGCCAGTTAG
PCK1-F	Primer	Human	GGCTGAAGAAGTATGACAAC
PCK1-R	Primer	Human	AATCCTCCTCTGACATCCA
SORD-F	Primer	Human	TTCAGCAAGTAGAGCAGAG
SORD-R	Primer	Human	GTCAGTAGCAGTGGAACC
si-LCN2#1	siRNA	Human	GCUGGGCAACAUUAAGAGUUAdTdT
si-LCN2#2	siRNA	Human	CCAGCAUGCUAUGGUGUUCUUdTdT

Figure S1: LCN2 mRNA expression in NCM460 cells after siRNA-mediated knockdown. Cells were transfected with si-NC, si-LCN2#1, or si-LCN2#2. LCN2 mRNA levels were measured by qRT-PCR at 48 hours post-transfection (n=3). *p < 0.05; **p < 0.01; ***p < 0.001; ****p < 0.0001. Related to Figure 7.

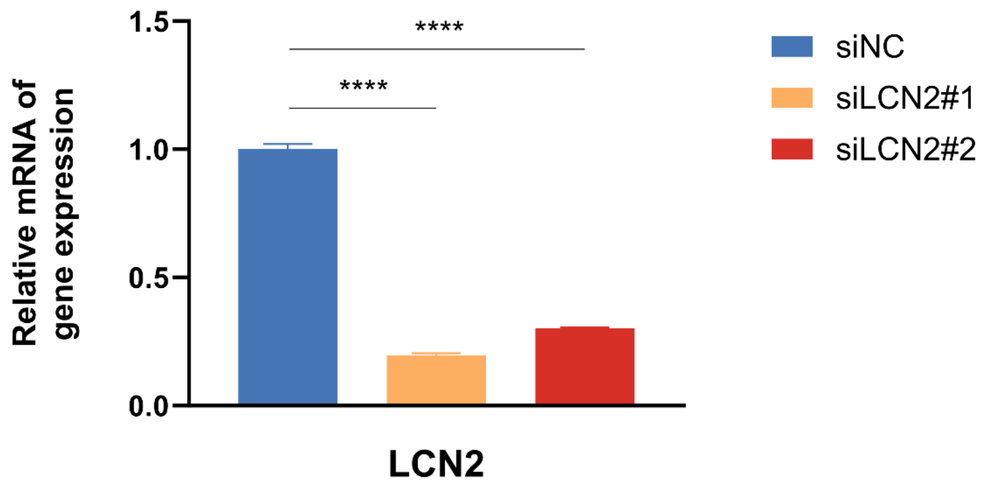

Figure S2: The raw images of western blotting. Related to Figure 7.
The images of western blotting in Fig 7B.

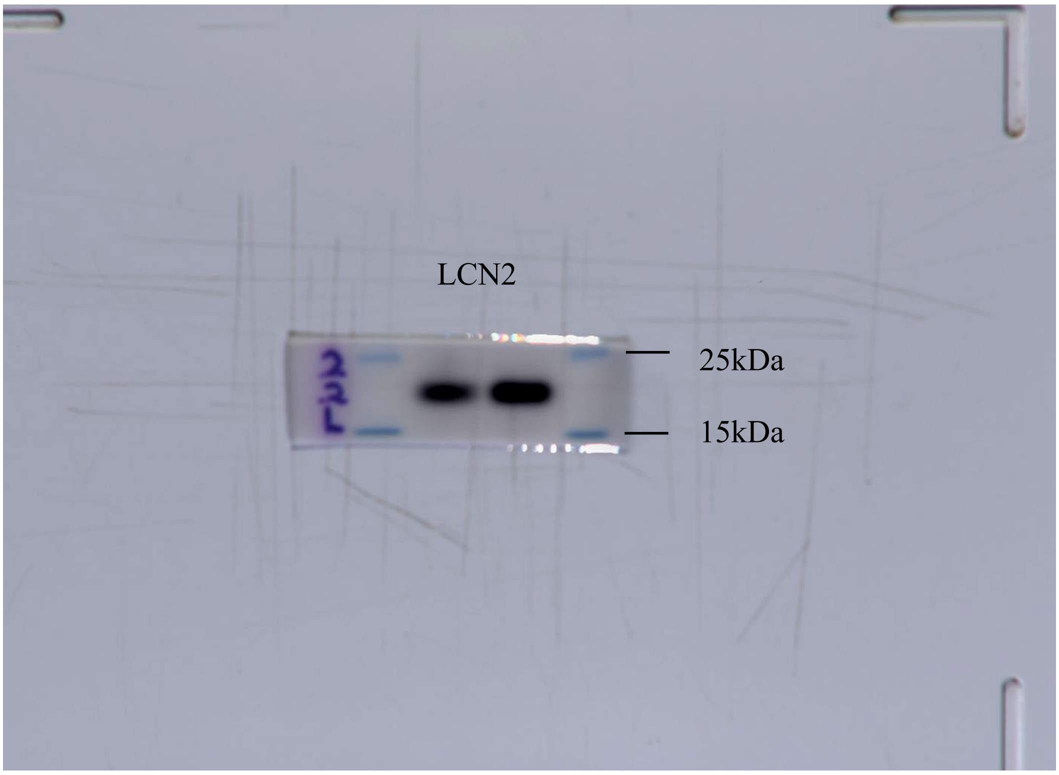

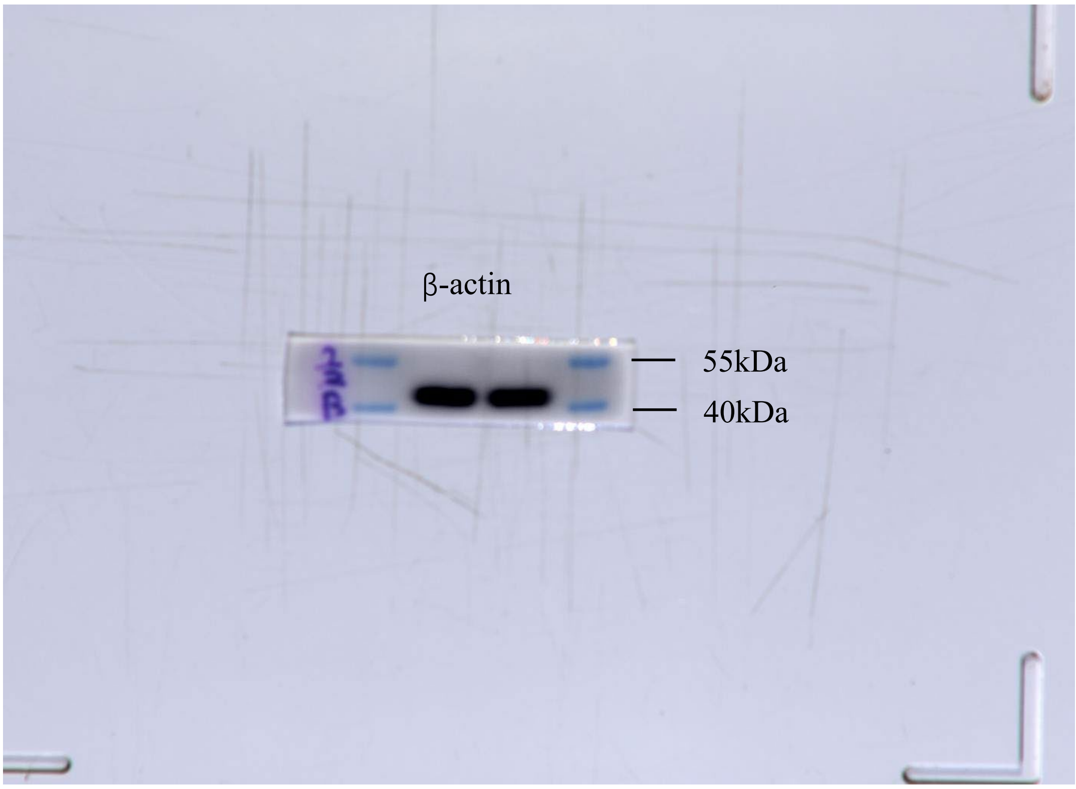

The images of western blotting in Fig 7C.

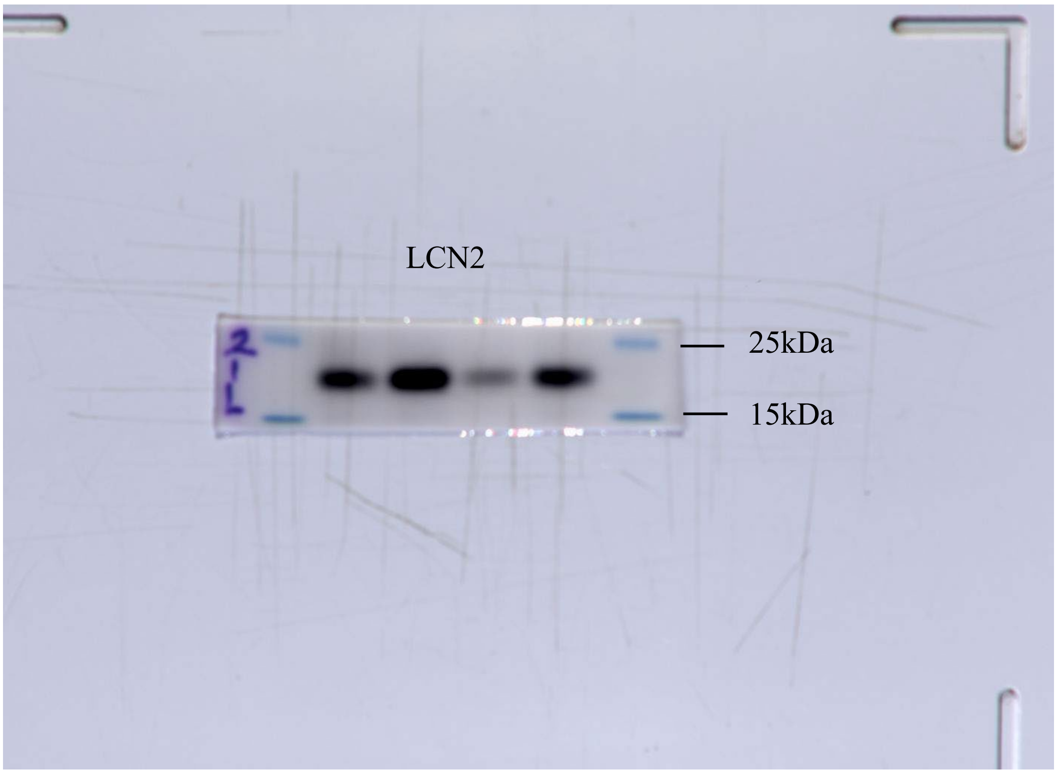

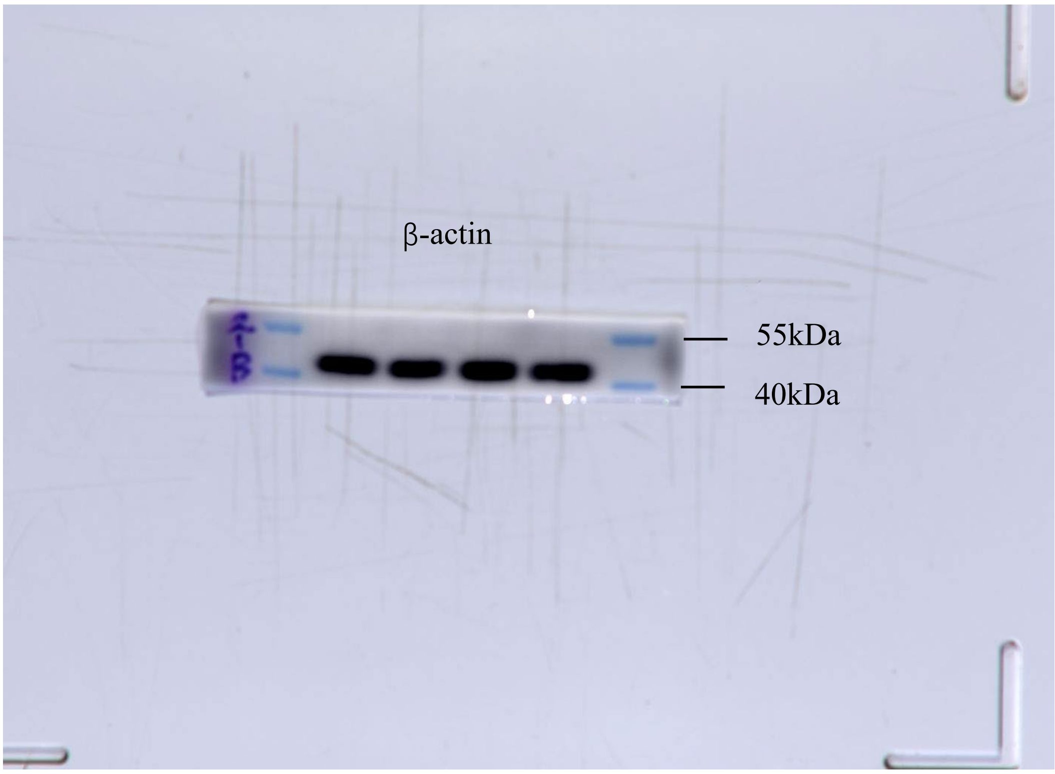

Supplement: Document S1. Figures S1 and S2 and Table S1 [file mmc1.pdf]
